# Performance of InterVA for assigning causes of death to verbal autopsies: multisite validation study using clinical diagnostic gold standards

**DOI:** 10.1186/1478-7954-9-50

**Published:** 2011-08-05

**Authors:** Rafael Lozano, Michael K Freeman, Spencer L James, Benjamin Campbell, Alan D Lopez, Abraham D Flaxman, Christopher JL Murray

**Affiliations:** 1 Institute for Health Metrics and Evaluation, University of Washington, 2301 Fifth Ave., Suite 600, Seattle, WA 98121, USA; 2 University of Queensland, School of Population Health, Brisbane, Australia

**Keywords:** Verbal autopsy, InterVA, validation

## Abstract

**Background:**

InterVA is a widely disseminated tool for cause of death attribution using information from verbal autopsies. Several studies have attempted to validate the concordance and accuracy of the tool, but the main limitation of these studies is that they compare cause of death as ascertained through hospital record review or hospital discharge diagnosis with the results of InterVA. This study provides a unique opportunity to assess the performance of InterVA compared to physician-certified verbal autopsies (PCVA) and alternative automated methods for analysis.

**Methods:**

Using clinical diagnostic gold standards to select 12,542 verbal autopsy cases, we assessed the performance of InterVA on both an individual and population level and compared the results to PCVA, conducting analyses separately for adults, children, and neonates. Following the recommendation of Murray et al., we randomly varied the cause composition over 500 test datasets to understand the performance of the tool in different settings. We also contrasted InterVA with an alternative Bayesian method, Simplified Symptom Pattern (SSP), to understand the strengths and weaknesses of the tool.

**Results:**

Across all age groups, InterVA performs worse than PCVA, both on an individual and population level. On an individual level, InterVA achieved a chance-corrected concordance of 24.2% for adults, 24.9% for children, and 6.3% for neonates (excluding free text, considering one cause selection). On a population level, InterVA achieved a cause-specific mortality fraction accuracy of 0.546 for adults, 0.504 for children, and 0.404 for neonates. The comparison to SSP revealed four specific characteristics that lead to superior performance of SSP. Increases in chance-corrected concordance are attained by developing cause-by-cause models (2%), using all items as opposed to only the ones that mapped to InterVA items (7%), assigning probabilities to clusters of symptoms (6%), and using empirical as opposed to expert probabilities (up to 8%).

**Conclusions:**

Given the widespread use of verbal autopsy for understanding the burden of disease and for setting health intervention priorities in areas that lack reliable vital registrations systems, accurate analysis of verbal autopsies is essential. While InterVA is an affordable and available mechanism for assigning causes of death using verbal autopsies, users should be aware of its suboptimal performance relative to other methods.

## Background

Verbal autopsy (VA) is increasingly being used in many monitoring, surveillance, and research settings [1-6]. In settings without complete vital registration and medical certification of death, VA provides one of the only methods for obtaining empirical information on cause of death patterns. The main strategy for assigning causes of death from data collected through a VA instrument is through physician-certified verbal autopsy (PCVA) [7-13]. Byass et al. proposed InterVA as an automated alternative to PCVA [14,15]. InterVA, now in edition 3.2 [16], has been applied in a number of research and demographic surveillance sites [14,17-25]. The method is based on the logic of Bayes' theorem. According to Bayes' theorem, prior views on the distribution of causes of death for a population are updated by each symptom response in the instrument. The probabilities of responding yes to an item conditional on the true cause of death have been developed through expert review panels.

Several studies have investigated the validity of InterVA as a tool for assigning causes of death [15,17,18]. A 2003 study analyzing 189 VA interviews in Vietnam found that, when considering all three possible causes assigned by the program, InterVA achieved over 70% concordance using PCVA as a comparator [14]. In another study that used InterVA to estimate AIDS deaths from 193 VA interviews in Ethiopia, the model correctly assigned 82% of AIDS deaths using hospital data as a gold standard [17]. Lastly, a study in Kenya that examined 1,823 VA interviews found 35% agreement between InterVA and physician review cause assignments [26]. The main limitation of these studies, as noted by several of the authors, is that they compare cause of death as ascertained through hospital record review or hospital discharge diagnosis with the results of InterVA. In low-resource and rural settings, where many of these studies have been conducted, the quality of the hospital diagnosis itself is often suspect. These studies provide information on the nominal association between hospital-assigned cause of death and InterVA, not true assessments of criterion validity where there is a gold standard cause of death. Further, comparison of InterVA with other published automated methods such as direct cause-specific mortality fraction (CSMF) estimation [27] or the Symptom Pattern Method [28] are limited by the reporting of different metrics in these studies.

The Population Health Metrics Research Consortium (PHMRC) provides an opportunity to assess the criterion validity of InterVA in a large multisite study. The PHMRC verbal autopsy study has been undertaken to develop a range of new analytical methods for verbal autopsy and to test these methods using data collected in six sites in four countries (Mexico, Tanzania, India, and the Philippines) [29]. The PHMRC study is unique both in terms of the size of the validation dataset (7,836 adult deaths, 2,075 child deaths, and 2,631 neonatal deaths) and the use of rigorously defined clinical diagnostic criteria for a death to be included in the study as a gold standard cause of death. Although the study was not originally designed to test the validity of InterVA, the study provides a unique opportunity to assess the performance of InterVA compared to PCVA and alternative automated methods for analysis.

## Methods

**Figure 1:**
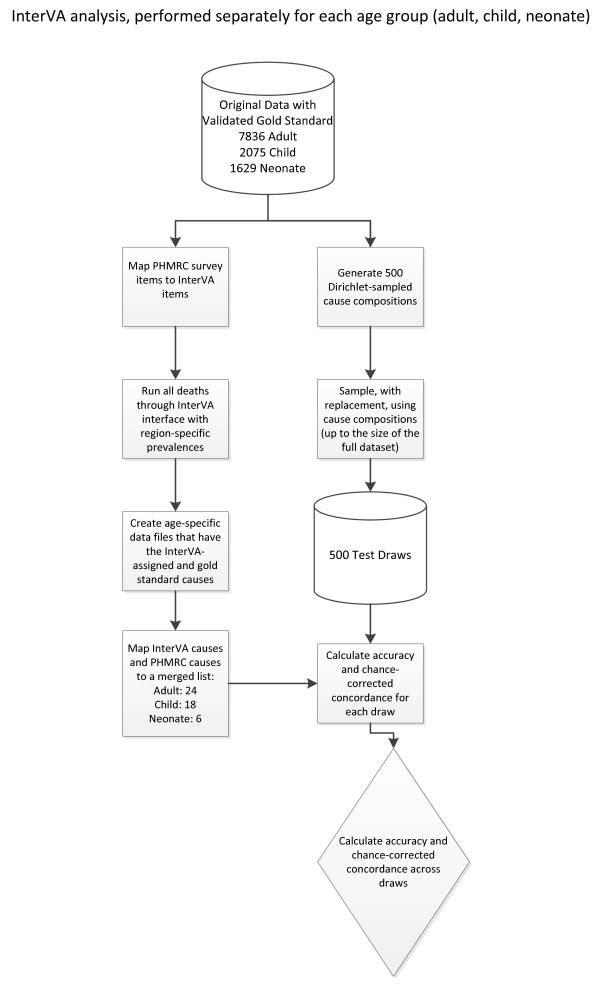
**Overview of analytical process**. This figure is a visual representation of the steps necessary for analysis, performed separately for each age group.

The design, implementation, and general descriptive results for the PHMRC gold standard VA validation study are described elsewhere [29]. The final study reports on 46 adult causes of death, 21 child causes of death, 10 neonatal causes of death, and stillbirths. Of note for this study, gold standard cause of death assignment was based on strict clinical diagnostic criteria defined prior to data collection - level 1 diagnostic criteria are stricter than level 2. Table 1 provides the number of adult, child, and neonatal deaths by cause (using the joint cause list described below). For the analysis in this paper, we present results pooling both level 1 and level 2 gold standard causes of death. We conduct and report on separate analyses for adult, child, and neonatal deaths. Figure 1 provides a visual representation of the overall approach of the methods.

**Table 1:** Number of deaths for adults, children, and neonates by cause

Adult causes	Deaths	Child causes	Deaths
Acute cardiac death	400	Chronic cardiac death	76

Chronic cardiac death	416	Chronic respiratory disease	12

Chronic respiratory disease	218	Diarrhea	256

Diabetes	414	Drowning	83

Diarrhea	228	HIV/AIDS	20

Disease of nervous system	49	Homicide	52

Drowning	106	Malaria	117

HIV/AIDS	501	Malignancy	28

Homicide	167	Measles	23

Kidney or urinary disease	413	Meningitis	99

Liver disease	313	Other acute infection	111

Malaria	100	Other digestive disease	48

Malignancy	1,090	Other injuries	171

Maternal Death	402	Other noncommunicable diseases	182

Other acute infection	263	Pneumonia/sepsis	678

Other digestive disease	166	Poisoning	18

Other injuries	464	Transport-related accident	92

Other noncommunicable diseases	200	Tuberculosis (pulmonary)	9

Pneumonia/sepsis	609	**Total**	**2075**

Poisoning	86		

Stroke	630	**Neonate causes**	**Deaths**

Suicide	124	Congenital malformation	250

Transport-related accident	202	Meningitis	6

Tuberculosis (pulmonary)	275	Perinatal asphyxia	461

**Total**	**7836**	Pneumonia/sepsis	250

		Preterm/small baby	662

		**Total**	**1629**

### Symptoms

InterVA version 3.2 is designed to have as input 106 items and yield predictions for 35 causes of death across all ages. The PHMRC data collection was based on a modification of the World Health Organization (WHO) instrument for VA, and Additional files 1, 2 and 3 list the PHMRC questions used to answer each InterVA item. Because InterVA does not interpret missing data, items not mapped from the PHMRC survey to the InterVA items were input as negative responses in InterVA. We extracted free text terms from open ended responses and coded them as dichotomous variables as described in the PHMRC study design paper [29]. Separate analyses were run with and without free text responses, but their inclusion had a negligible impact on the performance of the tool. In addition to the 106 symptom inputs, InterVA also uses priors for malaria and HIV/AIDS prevalence in the region of the deceased. We used regional malaria and HIV/AIDS prevalence as priors (see Additional file 4), but conducted a separate analysis in which we used the prevalence of a sample data draw as the priors. As we expected, using the regional prevalence was superior to using the draw prevalence.

### Cause lists

The PHMRC study included 46 causes for adults, 21 causes for children, 10 causes for neonates, and stillbirths. For each observation, InterVA predicts up to three causes of death from a list of 35 causes across all age groups. We have mapped the InterVA cause list and the PHMRC cause list into a set of mutually-exclusive, collectively-exhaustive cause categories for each age category. The details for this mapping are provided in Additional files 5, 6 and 7. The resulting joint cause lists contain 24 causes for adults, 18 causes for children, and six causes for neonates.

As mentioned above, InterVA can produce up to three potential causes for each death, and in some cases assigns deaths an indeterminate cause. Table 2 shows (by age group) the fraction of deaths to which InterVA assigned exactly one, two, or three causes, and the fraction deemed indeterminate. For modules reporting on only one cause assignment, we use the first cause of death to calculate chance-corrected concordance. We have also separately computed chance-corrected concordance using one, two, or all three InterVA cause assignments.

**Table 2:** Percent of deaths assigned to particular cause numbers by InterVA

	Exactly one assignment	Exactly two assignments	Exactly three assignments	Indeterminate
**Adult**	80.3%	16.1%	1.9%	1.8%

**Child**	76.7%	17.9%	1.9%	3.5%

**Neonate**	96.8%	2.6%	0.0%	0.5%

For calculating accuracy, indeterminate deaths were equally redistributed across the causes that InterVA had predicted. Redistribution of indeterminate causes across the other causes improves measured accuracy.

### Multiple validation test sets

As recommended by Murray et al. for validation studies [30], we vary the cause composition of the validation dataset by creating 500 test datasets. To do this, we first sample 500 distributions of CSMFs such that the sum of the CSMFs across causes equals 1.0. This is implemented by sampling from an uninformative Dirichlet distribution. We then randomly sample gold standard deaths with replacement to generate a test dataset with the desired CSMF composition. We then compute chance-corrected concordance and CSMF accuracy for each split (explained below). Because InterVA produces the same cause assignment for any given death, the deaths were run through the InterVA interface only once, and those cause assignments were used for the validation analysis.

### Metrics

Following the recommendations of Murray et al. [30], we assess the performance of InterVA compared to the gold standard using two types of metrics capturing the accuracy of individual death assignment and CSMF estimation. Assigning deaths to specific causes is assessed using cause-specific chance-corrected concordance and the average of cause-specific chance-corrected concordance across causes. As noted, to assess whether the second and third causes predicted for some deaths by InterVA improve performance, we also compute chance-corrected concordance incorporating the second and third cause assignments. Performance predicting CSMFs is assessed using CSMF accuracy, which is scaled from zero to one, where zero is the maximum possible error and one is no error in predicting CSMFs. The relationship between predicted CSMFs and true CSMFs across the 500 test datasets is summarized for each cause by performing a regression of true CSMFs on estimated CSMFs. Details on how to compute these metrics are provided in Murray et al. [30].

### Comparison to Simplified Symptom Pattern Method

Because we document poor performance of InterVA in comparison to PCVA [31], we have also compared InterVA to the Simplified Symptom Pattern (SSP) Method [28,32]. SSP is also based on Bayes' theorem; however, there are four key differences between InterVA and simplified SSP. First, the SSP Method develops Bayesian models for one cause compared to all other causes at a time, while InterVA considers all causes independently. Second, SSP uses the 40 most informative symptoms for each cause from the entire universe of all items in the survey, while InterVA is limited to the items that map to it (roughly one-third the number of inputs) and uses all of these symptoms (regardless of how informative they are). Third, SSP captures the interdependencies of the symptom responses, while InterVA considers each symptom individually. Finally, SSP uses empirical measurements of the probability of a symptom set conditional on the true cause captured in a training dataset, while InterVA uses expert opinion. Using the PHMRC data, we progressively change SSP to be more like InterVA and assess its performance using chance-corrected concordance and CSMF accuracy to understand which aspects of InterVA lead to poor performance. We analyzed three progressively changing permutations of the SSP Method to identify the effect each difference between SSP and InterVA had on the performances. First, we developed an SSP model for all causes at once rather than developing a model for each cause compared to all other causes at a time. Second, we restricted the universe of items available for SSP to only those used by InterVA. Third, we force SSP to assume that each item or symptom is independent of each other, as opposed to clustering different symptoms and developing probabilities of those combinations. Further details on SSP are available in Murray et al. [32].

## Results

### Performance assigning true cause to individual deaths

#### Across-cause results

Table 3 reports median chance-corrected concordances (across all causes) for one, two, and three cause assignments. The results are shown separately for all age groups, reporting on models with and without the inclusion of free text variables. Across all age groups and cause selections, the inclusion of free text variables at most increases chance-corrected concordance by 1.3%. The performance of InterVA, as measured by chance-corrected concordance, was comparable for adults and children using one cause selection (adults = 24.2%; children = 24.9%). However, the tool performed substantially worse for neonates, with a chance-corrected concordance of 6.3%.

**Table 3:** Median chance-corrected concordance (%) across causes for one, two, and three cause assignments (95% uncertainty interval [UI])

Age	Module	One cause	Two causes	Three causes
**Adult**	Free text	25.2 (25.1, 25.3)	25.1 (25.0, 25.1)	21.7 (21.6, 21.8)

	No free text	24.2 (24.1, 24.3)	24.0 (23.9, 24.1)	20.6 (20.5, 20.7)

**Child**	Free text	25.0 (24.7, 25.2)	22.5 (22.3, 22.7)	17.5 (17.3, 17.7)

	No free text	24.9 (24.7, 25.0)	21.4 (21.3, 21.7)	16.2 (16.1, 16.4)

**Neonate**	Free text	6.5 (6.2, 6.7)	-22.3 (-22.6, -22.0)	N/A

	No free text	6.3 (6.1, 6.5)	-22.8 (-23.0, -22.5)	N/A

In all three age groups, consideration of the second and third cause assigned by InterVA led to lower chance-corrected concordance, compared to consideration of only the first cause. This is largely due to the fact that InterVA rarely predicts more than one cause (at most 17% of cases).

**Figure 2:**
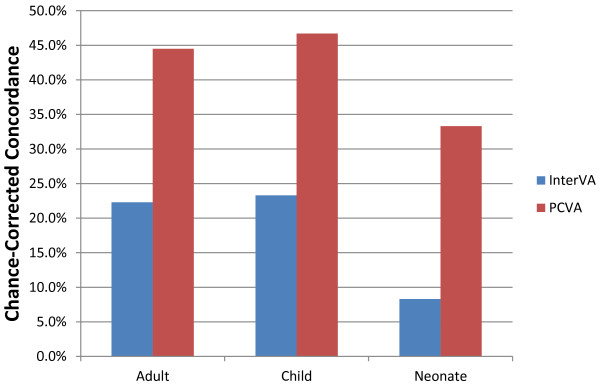
**Median chance-corrected concordance of InterVA and PCVA**. This figure compares the performance of InterVA with PCVA across 500 Dirichlet draws. PCVA performs better than InterVA for all age groups.

Figure 2 shows the comparison overall for adults, children, and neonates to PCVA as reported by Lozano et al. [31] for the PHMRC gold standard datasets. For all three age groups, InterVA has markedly lower chance-corrected concordances. Interestingly, the performances of InterVA and PCVA follow the same pattern, doing best in children by a small margin, followed by adults, and performing less well for neonates.

#### Cause-specific results

Additional file 8 shows the chance-corrected concordance, by cause, for adults, children, and neonates. These figures were calculated without the use of free text variables, and only considered the first InterVA cause assignment. These tables illustrate the distribution of InterVA's performance across causes.

For both adults and children, InterVA performed quite well for transport-related deaths; the chance-corrected concordances were 85.6% for adults and 95.7% for children. InterVA also did well on some other injuries, including its high chance-corrected concordance for poisoning (58.9%) and drowning (55.8%) in children. For adults, chance-corrected concordance was higher than 50% for homicide, liver disease, and tuberculosis, with nearly 50% for malignancy and maternal deaths. For children, in addition to the aforementioned injuries, InterVA had chance-corrected concordances of close to 50% for pneumonia/sepsis and HIV/AIDS. For neonates, the only cause with a chance-corrected concordance over 50% was perinatal asphyxia (77.4%).

While InterVA performed well for some causes such as these selected injuries, there were a number of causes that InterVA struggled to predict accurately. For adults, the lowest chance-corrected concordances were for disease of the nervous system (-4.3%), and the residual category other noncommunicable diseases (-4.0%). For children, InterVA struggled to accurately assign individual deaths for a number of categories. Similarly to adults, InterVA had poor performance with residual categories such as other acute infection and other digestive disease, with chance-corrected concordances of -5.9% for both causes. Chance-corrected concordance was also low for diseases that are rare in children, such as chronic cardiac death and malignancies. For neonates, InterVA did not perform well for a series of causes. Again, we saw the lowest chance-corrected concordance for the rarest cause (meningitis = -25.0%). Congenital malformation was another neonatal cause for which InterVA performed poorly, with a chance-corrected concordance of -12.9%.

### Performance estimating CSMFs

#### CSMF accuracy

Table 4 reports median CSMF accuracy (across all causes) for one, two, and three cause assignments. The results are shown separately for all age groups, reporting on models with and without the inclusion of free text variables. Across all age groups and cause selections, the inclusion of free text variables at most increases accuracy by 0.016. The performance of InterVA was comparable for adults and children, with an accuracy of 0.546 for adults and 0.504 for children. However, the tool performed substantially worse for neonates, with an accuracy of 0.404.

**Table 4:** Median CSMF accuracy across 500 Dirichlet draws, by age group and number of cause assignments (95% UI)

Age	Module	One cause	Two causes	Three causes
**Adult**	Free text	0.549 (0.542, 0.557)	0.555 (0.548, 0.563)	0.556 (0.548, 0.564)

	No free text	0.546 (0.539, 0.553)	0.554 (0.548, 0.560)	0.555 (0.549, 0.561)

**Child**	Free text	0.520 (0.513, 0.528)	0.503 (0.495, 0.511)	0.503 (0.496, 0.512)

	No free text	0.504 (0.496, 0.514)	0.487 (0.480, 0.494)	0.487 (0.482, 0.496)

**Neonate**	Free text	0.405 (0.392, 0.420)	0.409 (0.397, 0.425)	N/A

	No free text	0.404 (0.388, 0.419)	0.407 (0.393, 0.423)	N/A

In all three age groups, consideration of the second and third cause assigned by InterVA had a negligible effect on accuracy, with a maximum difference of 0.017. While the consideration of multiple cause assignments had a detrimental effect on chance-corrected concordance, that relationship was not seen for accuracy. This implies that, at the population level, the second and third cause assignments are as accurate as the first.

**Figure 3:**
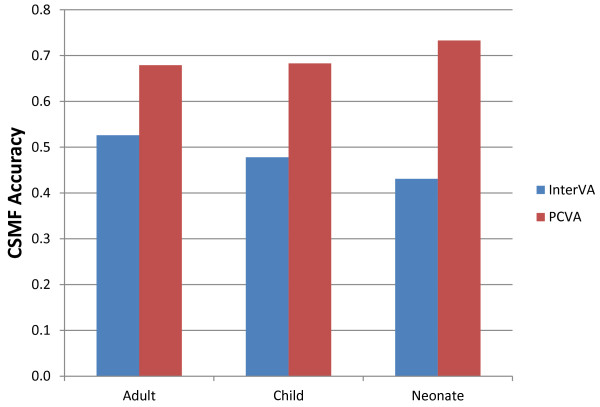
**Median CSMF accuracy of InterVA and PCVA**. This figure compares the performance of InterVA with PCVA across 500 Dirichlet draws. It shows a substantially better performance for PCVA than InterVA for all age groups.

Figure 3 summarizes CSMF accuracy for the three age groups and provides benchmark comparisons for PCVA as reported by Lozano et al. [31] for the same PHMRC gold standard database. In all age groups, CSMF accuracy is substantially lower than that observed for PCVA. Interestingly, InterVA performs better for older age groups, while PCVA performs better for younger age groups.

#### True versus estimated CSMFs

**Figure 4:**
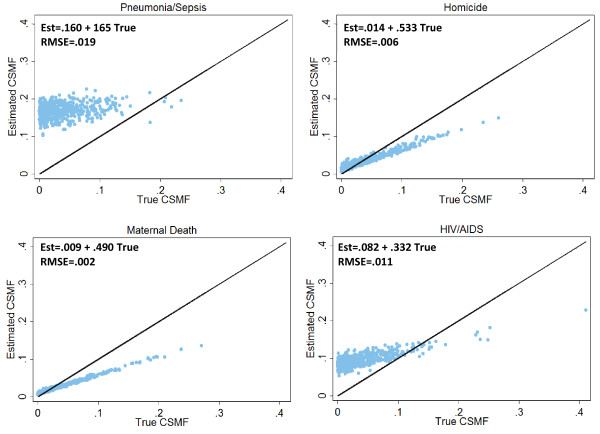
**Estimated versus true CSMFs**. This figure shows scatter plots of the estimated CSMF versus the true CSMF for pneumonia/sepsis, homicide, maternal death, and HIV/AIDS across 500 Dirichlet draws. It demonstrates the performance of InterVA for four causes of death as the cause fractions vary. Each graph shows the results from a regression of true CSMF on estimated CSMF, as well as the root mean squared error.

Figure 4 shows the results of regressing the true CSMF on the estimated CSMF for four selected adult causes (Additional file 9 shows the results for all causes for adults, children, and neonates). Each element of the output has a distinct implication for the relationship between true and estimated CSMFs. The ideal slope should be 1.00, such that a unit increase in the true CSMF corresponds to an equal unit increase in the estimated CSMF. The ideal intercept value is 0.00, and deviation from this provides information regarding the performance of the tool in populations with small cause fractions for that particular disease. Finally, the root mean squared error (RMSE) gives a measure of the uncertainty in the estimated CSMFs.

The causes selected for Figure 4 were chosen to demonstrate the differential performances of InterVA across causes. Both homicide and maternal death provide examples in which near-zero intercepts, 0.014 and 0.009 respectively, indicate good performance in sample populations with small cause fractions. However, in both instances, a slope that deviates substantially from 1.00 implies that InterVA will underestimate the proportion of these causes in populations where the disease is common. The low RMSEs (≤.006) indicate that the underestimation is consistent across different simulated populations, and may be amenable to a post hoc correction. Pneumonia/sepsis and HIV/AIDS provide examples in which the cause fractions are overestimated in draws with low cause fractions. With large intercepts, 0.160 and 0.082 respectively, InterVA predicts the presence of these conditions even if they are virtually absent in the population. Finally, higher RMSE values (> 0.01) suggest that correcting for this overestimation will be more difficult than correcting for the underestimation of homicide or maternal deaths.

### Comparison to SSP variants

**Figure 5:**
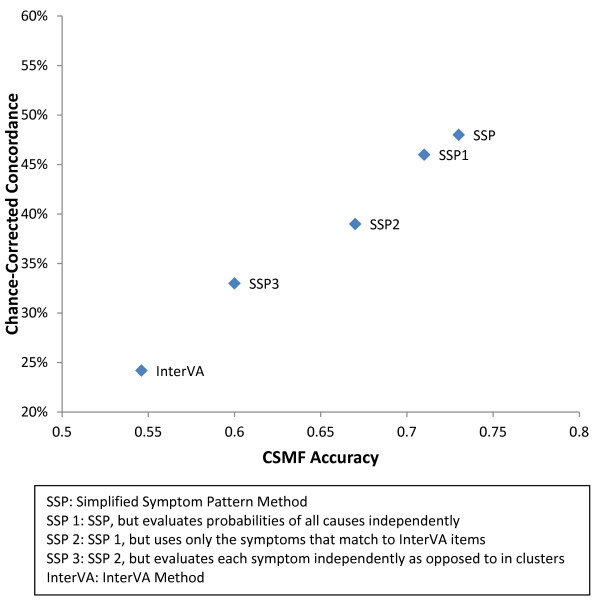
**Comparison of InterVA to variations of Simplified Symptom Pattern Method**. This figure shows the performances of four permutations of SSP versus InterVA for adults, considering one cause selection (excluding free text). It demonstrates the importance of different aspects of Bayesian methods.

Figure 5 shows a comparison of InterVA median chance-corrected concordance across causes with CSMF accuracy compared to three variants of SSP applied to the same dataset. Prior to modification, the SSP method had a chance-corrected concordance of 48% and an accuracy of 0.73. The first variant of SSP involved developing a model for all causes at once, rather than cause-by-cause models. This lowered chance-corrected concordance by 2% and accuracy by 0.02. The second variant further modified the methods by only using the survey questions that mapped to the InterVA survey. This lowered the chance-corrected concordance an additional 7% and lowered accuracy an additional 0.04. In addition to these changes, the third variation of SSP assumes the responses to each symptom are independent, as opposed to using clusters of symptoms that allow for correlation between items in response patterns. This method lowered the chance-corrected concordance by 6%, resulting in an overall chance-corrected concordance of 33% and an accuracy of 0.60. As SSP is modified to become more like InterVA, its performance both in terms of chance-corrected concordance and accuracy steadily declines.

**Figure 6:**
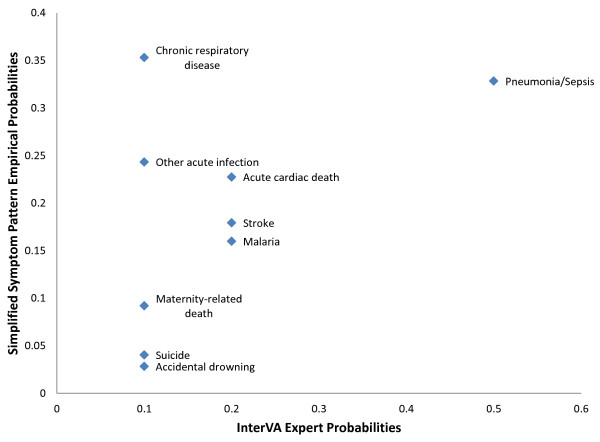
**Comparison of Simplified Symptom Pattern empirical probabilities and InterVA expert probabilities**. The scatter plot compares the probabilities of InterVA versus SSP for selected causes, given the symptom acute cough. This difference of posterior probabilities is partially responsible for the superior performance of SSP.

Figure 6 shows a comparison of selected empirical probabilities of SSP to the expert probabilities of InterVA for the symptom acute cough. This graph illustrates some of the differences in the prior probabilities of selected causes, which, based on the above analysis, may account for up to 8% chance-corrected concordance and 0.05 accuracy. Of note, InterVA tends to have higher probabilities than SSP for causes that are unrelated to cough (drowning, suicide, maternal death), while SSP has a higher probability for related causes such as infections and chronic respiratory disease.

## Discussion

This assessment of the performance of InterVA compared to gold standard cause of death assignment in a large multisite study shows an overall chance-corrected concordance of 24.2%, 24.9%, and 6.3% for adults, children, and neonates, respectively. At the level of estimating CSMFs, InterVA has a CSMF accuracy of 0.546 for adults, 0.504 for children, and 0.404 for neonates. Compared to PCVA, the performance of InterVA is much lower in terms of chance-corrected concordance, and it produces substantially larger errors in estimated CSMFs [31].

The poor performance of InterVA, given some published studies, is surprising. Not all studies, however, have reported good concordance. Oti et al. [33] compared InterVA on 1,823 deaths to physician review and found a chance-corrected concordance of 31.2%, which is not much higher than reported here - authors' calculations. One other validation study found a 33.3% chance-corrected concordance when comparing InterVA to physician review [14]. Two factors may account for the difference in the findings here compared with the more favorable studies. First, the PHMRC database is the first VA validation study where cause of death has been assigned using strict clinical diagnostic criteria and not medical record review or hospital diagnosis. The distinction is critical; in medical record review a chart may say myocardial infarction but not have documentation on how this diagnosis was made. In the PHMRC dataset, a death from myocardial infarction requires at least one of the following: cardiac perfusion scan, electrocardiogram changes, documented history of coronary artery bypass grafting or percutaneous transluminal coronary angioplasty or stenting, coronary angiography, and/or enzyme changes in the context of myocardial ischemia. Second, it is difficult to compare across previous studies because different metrics and results are reported for only one CSMF composition in the test data. Murray et al. report that findings can vary widely as a function of CSMF composition, and therefore metrics based on a single CSMF can be highly misleading [30].

Reporting chance-corrected concordance and regression results of CSMF true on CSMF estimated for each cause provides a framework for analyzing the strengths and weaknesses of InterVA. Clearly, the program is currently better suited to identify certain more obvious causes than other more complex ones. The program also has differential performances based on the cause fraction of each disease. This partly explains why different studies have shown different levels of accuracy for the program. InterVA could easily identify deaths with highly-probable symptoms such as road traffic injuries, but it struggled with less explicit causes such as infections. There also appeared to be some anomalous results from the program. For example, the program indicates that the probability of assigning drowning as a true cause is 0.99 if the respondent responded "yes" to the question "did s/he drown?" However, of the 117 adult deaths in which the respondent indicated that there was drowning, InterVA only assigned six of them "drowning" as the cause of death. We believe that this was the result of a coding error in the program. InterVA also tends to overpredict perinatal asphyxia in neonates. While we are less confident why this is, we believe that it is a notable shortcoming of the program. We hope that the cause-specific results can be used to better inform expert priors for future Bayesian methods.

The analysis of InterVA compared to the other Bayesian automated approach, Simplified Symptom Pattern, also provides a clear indication of why InterVA is not working well. The analysis of SSP variants designed to approximate InterVA show that four factors contribute to better results using SSP: use of interdependencies in the symptom responses, the use of all the items in the WHO or PHMRC instrument rather than just the 106 items in InterVA, the use of empirical probabilities of symptoms conditional on the true cause rather than expert judgment, and finally the technical advantage of developing models for each cause relative to other causes rather than all causes independently [32]. Moving to empirical probabilities improved chance-corrected concordance by 4%, capturing the interdependencies of some items added another 6%, and expanding from the InterVA item list to the full item list added another 7%. The progressive improvement in the performance of the SSP variants provides an understanding of how the limitations of the implementation of Bayes' theorem in InterVA contribute to its poor performance.

There are several limitations of this study. First, because the InterVA and PHMRC cause lists had to be merged to a joint cause list, InterVA was essentially challenged to predict causes that it was not built to identify (such as specific types of injuries). Conversely, there are a number of causes for which InterVA may predict very well that were not included in the study (such as malnutrition in children). InterVA could in theory perform well for these causes, which would have increased its average chance-corrected concordance. Note that the cause list used for the assessment of PCVA performance was slightly longer, so the InterVA performance may have been slightly exaggerated [31]. Second, there were a number of InterVA items that were not mapped to the PHMRC survey (17 adult questions, 32 child questions, and 30 neonatal questions). Inclusion of these items would likely improve performance of the tool. Third, InterVA predicted deaths in some age groups for causes that largely belong to other age groups. For example, it predicted preterm/small baby as a child cause and malnutrition as an adult cause. These deaths were assigned to the residual other category. This practice also may have exaggerated InterVA accuracy.

The contribution of this study is the use of gold standard cases for the validation of InterVA. The aforementioned studies only provide information on the relationship between InterVA and hospital- assigned or physician-reviewed cause of death. This study provides a direct comparison of InterVA to gold standard verified causes of death. It is also important to note that this study is considering the performance of InterVA in a diverse cultural and epidemiological context. However, further analysis from each of the sites will provide specific results about the performance of InterVA in each of the countries included in the PHMRC study.

## Conclusions

This study demonstrated both the strengths and weaknesses of InterVA as a method of assessing both individual-level and population-level causes of death. For the first time, the use of gold standards for validation illustrates the performance of the tool in diverse settings. To date, InterVA has proven popular with some users because it is automated and can reduce the cost of VA analysis and speed up data processing. InterVA does not use free text items and implicitly encourages users to use structured instruments that may also lead to savings and efficiencies in data processing. The relative computational simplicity of InterVA also means that it can work in a variety of settings without access to more sophisticated computational power that might be required for some empirically-derived methods. Additionally, InterVA is not linked to a specific VA instrument, which is both a strength and a weakness. The strength is that, in principle, it can be used to analyze data collected historically with different or more limited instruments. The weakness, however, is that much of the salient information collected in the WHO or PHMRC instruments are not used. Further, because it is not tied to an instrument, the InterVA items are defined in medical terms and are not actually mapped to particular questions that can be asked of households. Such ambiguity stems from the specification of the InterVA variables as medical terms rather than VA instrument items.

These advantages come at a substantial decrement in performance compared to PCVA. Fortunately, other automated options for the analysis of VA data have the same advantages but have validated performance equal to or better than PCVA, such as the Tariff Method, SSP, and machine learning [32,34,35]. Given the widespread use of VA for understanding the burden of disease and setting health intervention priorities in areas that lack reliable vital registrations systems, accurate analysis of VAs is essential. Until InterVA is substantially revised, users should carefully consider the use of alternative automated approaches for the analysis of VA data.

## Abbreviations

CSMF: cause-specific mortality fraction; PCVA: physician-certified verbal autopsy; PHMRC: Population Health Metrics Research Consortium; RMSE: root mean squared error; SSP: Simplified Symptom Pattern Method; VA: verbal autopsy.

## Competing interests

The authors declare that they have no competing interests.

## Authors' contributions

RL, ADL, ADF, and CJLM designed the study. MKF and SLJ performed the statistical analyses. BC conducted the literature review and participated in the analysis. RL, MKF, and CJLM drafted the manuscript and approved the final version. RL accepts full responsibility for the work and the conduct of the study, had access to the data, and controlled the decision to publish. All authors have read and approved the final manuscript.

## Supplementary Material

Additional file 1 **Mapping between InterVA input questions and PHMRC survey questions for adults**. Click here for file

Additional file 2 **Mapping between InterVA input questions and PHMRC survey questions for children**. Click here for file

Additional file 3 **Mapping between InterVA input questions and PHMRC survey questions for neonates**. Click here for file

Additional file 4 **Regional prevalence priors used for InterVA**. Click here for file

Additional file 5 **Mapping between InterVA causes and PHMRC causes for adults**. Click here for file

Additional file 6 **Mapping between InterVA causes and PHMRC causes for children**. Click here for file

Additional file 7 **Mapping between InterVA causes and PHMRC causes for neonates**. Click here for file

Additional file 8 **Chance-corrected concordance (%) for adult, child, and neonatal causes across 500 Dirichlet draws (excluding free text, one cause selection)**. Click here for file

Additional file 9 **Results of regressing true CSMFs on estimated CSMFs for adult, child, and neonatal causes (excluding free text, one cause selection)**. Click here for file
